# Synthesis, Structural Characterization and Biological Activity of Novel Cyclohexane-1,3-dione Ligands and Their Metal Complexes

**DOI:** 10.3390/molecules20059309

**Published:** 2015-05-21

**Authors:** Nevin Turan, Hanifi Körkoca, Ragıp Adigüzel, Naki Colak, Kenan Buldurun

**Affiliations:** 1 Department of Chemistry, Faculty of Arts and Sciences, Muş Alparslan University, 49250 Muş, Turkey; E-Mail: k.buldurun@alparslan.edu.tr; 2 Nursing Department, School of Health, Muş Alparslan University, 49250 Muş, Turkey; E-Mail: h.korkoca@alparslan.edu.tr; 3 Department of Chemical Engineering, Faculty of Engineering, Tunceli University, 62000 Tunceli, Turkey; E-Mail: radiguzel@tunceli.edu.tr; 4 Department of Chemistry, Faculty of Arts and Sciences, Hitit University, 19100 Çorum, Turkey; E-Mail: nakicolak@hitit.edu.tr

**Keywords:** cyclohexane-1,3-dione, antibacterial activity, metal complexes, mass spectra, spectroscopic characterization

## Abstract

Some new Zn(II) and Cu(II) complexes [Cu(L^1^)(OAc)_2_]∙H_2_O, [Cu(L^1^)(NO_3_)H_2_O]∙NO_3_∙3.5H_2_O, [Zn(L^1^)(NO_3_)_2_]∙4.5H_2_O, [Zn(L^1^)(OAc)_2_(H_2_O)_2_]∙3H_2_O, [Cu_2_(L^2^)(OAc)_4_]∙2H_2_O∙2DMF, [Cu(L^2^)_2_]∙2NO_3_∙1.5DMF∙H_2_O, [Zn(L^2^)_2_(NO_3_)_2_]∙DMF and [Zn_2_(L^2^)(OAc)_4_(H_2_O)_4_]∙5H_2_O; L^1^ = 2-[2-(2-methoxyphenyl)hydrazono]cyclohexane-1,3-dione and L^2^ = 2-[2-(3-nitrophenyl)hydrazono]cyclohexane-1,3-dione were synthesized and characterized by IR, ^1^H-NMR,^13^C-NMR and ultraviolet (UV-Vis.) spectroscopy, elemental analysis, magnetic susceptibility, mass spectrometry and thermogravimetry-differential thermal analysis (TGA-DTA). The synthesized ligands and their complexes were tested for antibacterial activity against *Escherichia coli* ATCC 25922, *Enterococcus faecalis* ATCC 29212, *Staphylococcus aureus* ATCC 25923, and *Salmonella typhimurium* CCM 583. Some of complexes showed medium-level antibacterial activity against the test bacteria compared with ampicillin.

## 1. Introduction

Cyclohexane-1,3-dione and its derivatives are important building blocks. The cyclohexane-1,3-dione skeleton is a characteristic molecular fragment common for a class of natural and synthetic herbicides and drugs inhibiting 4-hydroxyphenylpyruvate deoxygenase [1,2]. The inhibitory properties of this class of compounds are a result of their ability to chelate the ferrous ion in the active site of the enzyme.

Arylhydrazones of mono-, di- and triketones as well as cyclic 1,3-diones *viz*., cyclohexane-1,3-dione have been reported in the literature. Such compounds have been extensively used as precursors of potential anti-diabetic drugs [3]. Hydrazones are compounds whose molecules contain the C=N-NH- triatomic linkage. The biological, chemical and industrial versatility of hydrazones and their complexes continue to attract considerable attention [4,5]. The role of hydrazones in treating tuberculosis is well known. Substituted hydrazones have established spasmolytic activity, activity against leukemia, and sarcomas [6]. Also they possess anti-inflammatory, analgesic, antipyretic, antibacterial and antitumor activities [7].

**Figure 1:**
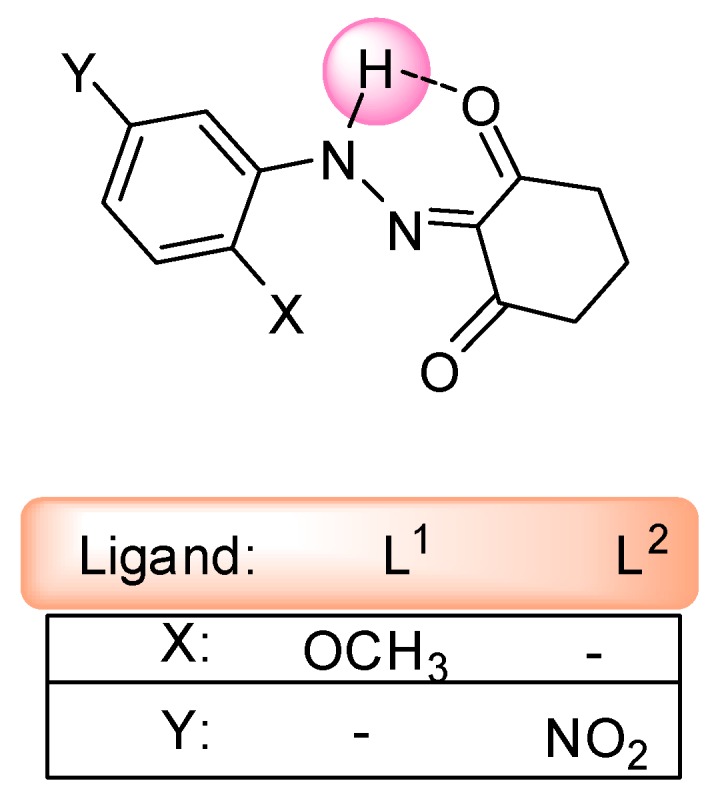
Structures of the ligands L^1^ and L^2^.

In order to investigate the relationship of ligands L^1^ and L^2^ and their complexes with their biological activities, we synthesized two new ligand, 2-[2-(2-methoxyphenyl)hydrazono]cyclohexane-1,3-dione (L^1^) 2-[2-(3-nitrophenyl)hydrazono]cyclohexane-1,3-dione (L^2^). Then, their Zn(II) and Cu(II) complexes were synthesized by reaction of Zn(NO_3_)_2_∙6H_2_O, Cu(NO_3_)_2_∙2H_2_O, Zn(OAc)_2_∙2H_2_O and Cu(OAc)_2_∙H_2_O, respectively. The structure of the ligands is shown in Figure 1. Finally, all of the synthesized compounds were tested for their antibacterial activities against *Escherichia coli* ATCC 25922 (non-β-lactamase producing), vancomycin susceptible *Enterococcus faecalis* ATCC 29212, methicillin susceptible *Staphylococcus aureus* ATCC 25923, and *Salmonella typhimurium* CCM 583.

## 2. Results and Discussion

### 2.1. Infrared Spectra

IR absorptions of both L^1^ and L^2^ and their metal complexes along with assignments are summarized in the experimental section. All complexes are air stable and insoluble in most organic solvents and water, but freely soluble in coordinating solvents such as pyridine, DMF or DMSO. All have higher melting or decomposition points than the parent ligands.

In the IR spectrum of L^1^ the characteristic peaks are seen at 2955–3500 cm^−1^ ν(NH), 1280 cm^−1^ ν(OCH_3_), 1696, 1671 cm^−1^ ν(C=O), and 1505 cm^−1^ ν(C=C) [8,9,10,11]. The L^1^ shows a broad NH band at 3343 cm^−1^ due to the hydrazone NH group. The position and broadness of the band are indicative of intramolecular hydrogen bonding between the hydrazone proton and the carbonyl oxygen. The hydrazone can coordinate to transition metals either in the enolic form (N=N-C=C-OH) or in ketonic form (HN-N=C-C=O). In L^1^ and L^2^ the characteristic hydrazone group >CONH- peaks indicate that the ligand exists in the keto form in the solid state. This NH band shifted upon complexation of the ligand with metals due to coordination of the hydrazone proton in [Cu_2_(L^2^)(OAc)_4_]∙2H_2_O∙2DMF and all of the Zn(II) complexes [12,13].

The strong bands at 1696, 1671 and 1686 cm^−1^, in the spectra of L^1^ and L^2^, respectively, may be assigned to ν(C=O). In the spectra of all complexes, except those of [Zn(L^1^)(NO_3_)_2_]∙4.5H_2_O, [Zn(L^1^)(OAc)_2_(H_2_O)_2_]∙3H_2_O and [Zn(L^2^)_2_(NO_3_)_2_]∙DMF, these bands are found to be shifted to lower frequency, indicating the participation of the carbonyl oxygen in coordination. Peaks at 1671, 1686 cm^−1^ ν(C=O), 1626, 1633 cm^−1^ ν(C=O….H) and 1592 cm^−1^ ν(C=N) also support the H-bonded hydrazone structure in the solid state [14]. In the [CuL^1^(NO_3_)H_2_O]∙NO_3_∙3.5H_2_O and [Cu(L^2^)_2_]∙2NO_3_∙1.5DMF∙H_2_O complexes, bands appearing at 1384 and 1382 cm^−1^, respectively, suggest the presence of ionic nitrate in the complexes [14].

In the IR spectrum of L^2^, the characteristic peaks are at 1686 and 1353 cm^−1^, which are assigned to ν(C=O) and ν(NO_2_), and at 1614 cm^−1^ which is assigned to the ν(C=N) group [15,16]. In addition, the spectrum of the ligand L^2^ shows broad bands in the 3500–3100 cm^−1^ region, which may be assigned to a ν(NH) band. In the IR spectra of L^2^, the bands assigned to ν(C=O) and ν(C=N) are shifted by ±16–35 cm^−1^ in the spectra of complexes, indicating coordination through the exocyclic carbonyl oxygen and C=N nitrogen of L^2^. In the spectra of the metal complexes of the L^2^, the band at 1353 cm^−1^ for the NO_2_ group did not shift [17], showing that in these complexes the NO_2_ group did not participate in complex formation. In the IR spectra of the complexes, ν(OCH_3_) remains unmodified, indicating that the methoxy group is also not involved in the coordination.

The infrared spectra of all complexes, except that of [Zn(L^2^)_2_(NO_3_)_2_]∙DMF complex, exhibited intense broad bands at 3560–3311 cm^−1^ that are attributed to ν(OH) of the lattice or coordinated water molecules, while the ν(H_2_O) bands observed at approximately 876–764 cm^−1^ are assigned to coordinated water molecules [18,19,20]. The spectra of the complexes show a few new absorption bands in the 491–455 cm^−1^ and 585–510 cm^−1^ ranges, assigned to ν(M-N) and ν(M-O), respectively.

The complexes [Cu(L^1^)(NO_3_)H_2_O]∙NO_3_∙3.5H_2_O, [Zn(L^1^)(OAc)_2_(H_2_O)_2_]∙3H_2_O and [Zn_2_(L^2^)(OAc)_4_(H_2_O)_4_]∙5H_2_O show a broad band at 3468–3311 cm^−1^ indicating the coordination of water to the metal ion. From the IR results, it may be concluded that the L^1^ and L^2^ ligands are bi- or tridentate and coordinate with the metal ions through the carbonyl oxygen, hydrazone nitrogen and/or acetic acid oxygen atoms.

The ν(N-N) bands of the L^1^ and L^2^ ligands at 1025, 1010 cm^−1^ are found to be shifted to higher energies (1041–1020 cm^−1^) in the spectra of the complexes, indicating coordination via the nitrogen atoms. This is confirmed by bands in the range of 491–455 cm^−1^, which have been assigned to the ν(Zn-N) ν(Cu-N) bands [21].

The IR spectra of the [Cu_2_(L^2^)(OAc)_4_]∙2H_2_O∙2DMF, [Cu(L^2^)_2_]∙2NO_3_∙1.5DMF∙H_2_O and [Zn(L^2^)_2_(NO_3_)_2_]∙DMF complexes show strong bands around 1660 cm^−1^ assigned to a ν(C=O) vibration of the DMF molecules [22]. The IR spectra of [Cu(L^1^)(OAc)_2_]∙5H_2_O, [Zn(L^1^)(OAc)_2_(H_2_O)_2_]∙3H_2_O, [Cu_2_(L^2^)(OAc)_4_]∙2H_2_O∙2DMF and [Zn_2_(L^2^)(OAc)_4_(H_2_O)_4_]∙5H_2_O show two new bands at 1555 and 1384 cm^−1^ assignable to the ν_as_(C–O) and ν_s_(C–O) bands of the acetate group [23,24], respectively, which are consistent with monodentate acetate coordination. The IR spectra of the [Cu(L^1^)(NO_3_)H_2_O]∙NO_3_∙3.5H_2_O, [Zn(L^1^)(NO_3_)_2_]∙4.5H_2_O and [Zn(L^2^)_2_(NO_3_)_2_]∙DMF complexes show strong bands at 1385 and 1285 cm^−1^ assigned to ν_as_(NO_3_^−^) and ν_s_(NO_3_^−^), respectively, indicating the presence of terminally bounded monodentate nitrate groups [25,26].

### 2.2. ^1^H-NMR and ^13^C-NMR Spectra

The ^1^H-NMR spectra of L^1^, L^2^ and their Zn(II) complexes were recorded in dimethylsulfoxide (DMSO-d_6_) solution using tetramethylsilane (TMS) as internal standard. The ^1^H-NMR spectrum of L^1^ shows a singlet at 4.00 ppm that may be assigned to the protons of the methoxy group, a singlet at 15.00 ppm that may be assigned to the proton of the NH group, a multiplet at 7.20–7.80 ppm that may be assigned to the aromatic protons and another at 2.00–2.70 ppm that may be assigned to the hexane ring protons. Similar peaks are observed for the ligand L^2^. The ^1^H-NMR spectrum of the diamagnetic Zn(II) complexes shows almost the same values as the ligands. The signals due to the -NH proton are shifted downfield in the spectra of the Zn(II) complexes, indicating the coordination of the ligand through the nitrogen of the -NH groups to the metal ions. The hydrogen bonding decreases the electron density around the proton and thus moves the proton absorption to a lower field [27]. In addition, H_2_O and acetate proton signals are seen in NMR spectra of complexes.

### 2.3. Electronic Spectra and Magnetic Properties

The electronic spectra of L^1^, L^2^ and their metal complexes were recorded in 3 × 10^–3^ molar DMF solutions in the range from 200 to 800 nm. The electronic spectrum of the ligands shows broad bands at 218, 242 and 316–393 nm; the first two absorption bands may be assigned to n→π* and π→π* transitions of >C=O and >C=N moieties [28], the third absorption band may be due to high-intensity charge transfer transitions.

The [Cu(L^1^)(OAc)_2_]∙5H_2_O complex shows square-planar geometry. It shows intense absorption bands in the 254–269 and 414 nm range which can be assigned to the π→π* transition of the C=N or C=O groups and charge-transfer bands, respectively. The electronic spectrum of the complex shows an absorption band in the range 580 nm, attributed to ^2^T_2g_→^2^E_g_ transition suggesting a square-planar geometry [29].

The electronic absorption spectrum of the [Cu(L^1^)(NO_3_)H_2_O]∙NO_3_∙3.5H_2_O complex in DMSO solution shows three bands at 254–295, 353, 393 and 428–450 nm, assignable to the ^2^B_1g_→^2^B_2g_, ^2^B_1g_→^2^E_g_, and ^2^B_1g_→^2^A_1g_ transitions and two intra-ligand charge transfer bands. These data and the magnetic moment value of 1.72 B.M. suggest square-planar geometry around Cu(II) [30,31].

Square planar geometry is suggested for [Cu_2_(L^2^)(OAc)_4_]∙2H_2_O∙2DMF from the presence of bands at 600 and 454 nm for the complex. These bands are assigned to the ^2^B_1g_→^2^A_1g_ and ^2^B_1g_→^2^E_g_ transitions, while the second band is due to charge transfer. The magnetic moment values for the Cu(II) complex are normal and lower (1.66 B.M.) than expected 1.7–2.2 B.M. for one unpaired electron [32,33].

The electronic spectrum of Cu(II) complex shows bands in the regions of 254, 376 and 470 nm which may be assigned to the ^2^B_1g_→^2^A_1g_ transitions in a square-planar geometry. The magnetic moment value of [Cu(L^2^)_2_]∙2NO_3_∙1.5DMF∙H_2_O complex is 2.22 B.M. [34].

The electronic spectra of the Zn(II) complexes show only a high-intensity band at 357–425 cm^−1^ assigned to ligand-metal charge transfer.

### 2.4. Thermal Studies

The thermal stability of the ligands and complexes is investigated using thermogravimetric analysis. The TGA curves are obtained at a heating rate of 10 °C∙min^−1^ under a nitrogen atmosphere in a temperature range of 25.0–800.0 °C. The results obtained are in good agreement with the theoretical formula suggested from the elemental analyses. The thermal data are summarized in Table 1.

The [Cu_2_(L^1^)_2_(AcO)_2_]∙5H_2_O complex is stable up to 95 °C and its decomposition starts at this temperature. A 16.92(17.39%) weight loss is observed at 195 °C corresponding to five moles of water of crystallization. The DTA curve of the complex shows one endothermic peak at 350.50–360.00 °C. For the Cu(II) complex, the DTA curve shows an endothermic process around 350.50 °C that corresponds to the rupture of the coordinated bond and simultaneous melting of the complex [35].

The [Cu(L^1^)(NO_3_)H_2_O]∙NO_3_∙3.5H_2_O complex was stable up to 85 °C and its decomposition started at this temperature. The first decomposition stage at 160 °C is attributed to the removal of 3.5 moles of water of crystallization and 1 mole of nitrate ion with a 24.62(24.29%) loss. The second weight loss stage of 234.48–494.55 °C corresponds to the removal of the H_2_O, NO_3_^–^ and C_6_H_6_O_2_N as a fragment of the ligand decomposition. The DTA curve of the Cu(II) complex shows two endothermic peaks at 150.10 °C, 380.50 °C and one exothermic peak at 485.25 °C.

The [Zn(L^1^)(NO_3_)_2_]∙4.5H_2_O complex decomposed in three stages. In the first stage, loss of 4.5 moles of water of crystallization and in the second stage, loss of two moles of NO_3_^–^ and C_13_H_14_O_2_N_2_ groups resulted. In the third stage, the complex decomposed to ZnO. The DTA curve of the complex shows two endothermic peaks at 450.00–470.20 °C. For the Zn(II) complex, these peaks corresponds to simultaneous melting of the complex [35].

The [Zn(L^1^)(OAc)_2_(H_2_O)_2_]∙3H_2_O complex decomposed in two stages. The first is due to removal of the crystallization water molecule with a weight loss of 18.76(18.83%). The second weight loss is associated with the removal of two moles of water as well as 2C_2_H_3_O_2_^−^ from the ligand [36]. The DTA curve of the Zn(II) complex shows two endothermic peaks at 280.10 °C, 330.50 °C and one exothermic peak at 420.25 °C.

**Table 1:** Proposed decomposition steps and the respective mass losses of ligands (L^1^ and L^2^) and their complexes.

Equations	Temperature (°C)	% Loss in Weight % Found(% Calculated)	Decomp. Products
[Cu(L^1^)(OAc)_2_]∙5H_2_O			
C_17_H_30_N_2_O_12_Cu	95.50–250.00	16.92(17.39)	5 H_2_O
C_17_H_20_N_2_O_7_Cu	250.00–514.29	40.00(40.36)	2 OAc^−^, C_6_H_5_N
C_7_H_9_NO_3_Cu	514.29–650.00	24.62(23.96)	C_6_H_6_NO_2_
CH_3_OCu	650–Cont.		
[Cu(L^1^)(NO_3_)H_2_O]∙NO_3_∙3.5H_2_O			
C_13_H_16_N_4_O_10_Cu∙3.5H_2_O	85.25–234.48	24.62(24.29)	3.5 H_2_O, NO_3_^−^
C_13_H_16_N_3_O_7_Cu	234.48–494.55	40.00(39.64)	H_2_O and NO_3_^−^,C_6_H_6_O_2_N
C_7_H_8_NOCu	494.55–Cont.		
[Zn(L^1^)(NO_3_)_2_]∙4.5H_2_O			
C_13_H_14_N_4_O_9_Zn∙4.5H_2_O	76.19–457.14	15.39(15.69)	4.5 H_2_O
C_13_H_14_N_4_O_9_Zn	457.14–495.23	67.69(68.55)	2NO_3_^−^, C_13_H_14_O_2_N_2_
ZnO	495.23–Cont.		
[Zn(L^1^)(OAc)_2_(H_2_O)_2_]∙3H_2_O			
C_17_H_30_N_2_O_12_Zn	80.32–284.75	18.76(18.83)	3 H_2_O
C_17_H_24_N_2_O_9_Zn	284.75–423.50	29.23(29.65)	2 H_2_O and 2 C_2_H_3_O_2_^−^
C_13_H_14_N_2_O_3_Zn	423.50–Cont.		
[Cu_2(_L^2^)(OAc)_4_]∙2H_2_O∙2DMF			
C_26_H_41_N_5_O_16_Cu_2_	180.12–240.32	23.08(22.58)	2 H_2_O and 2DMF
C_20_H_23_N_3_O_12_Cu_2_	240.32–285–340	29.23(29.27)	4 OAc^−^
C_12_H_11_N_3_O_4_Cu_2_	285–340		
[Cu(L^2^)_2_]∙2NO_3_∙1.5DMF∙H_2_O			
C_24_H_24_N_8_O_15_Cu∙1.5DMF	100.05–280.14	24.62(24.79)	H_2_O, 1.5 DMF, 2 NO_3_^−^
C_24_H_22_N_6_O_8_Cu	280.14–600.00	58.46(58.55)	C_24_H_22_N_6_O_7_Cu
CuO			
[Zn(L^2^)_2_(NO_3_)_2_]∙DMF			
C_24_H_22_N_7_O_16_Zn	160.53–241.20	9.23(9.30)	DMF
C_21_H_15_N_6_O_15_Zn	241.20–277.55	44.61(44.10)	2 NO_3_^−^, C_12_H_14_N_2_O_2_
C_9_HN_2_O_7_Zn	277.55–458.33		
[Zn_2_(L^2^)(OAc)_4_(H_2_O)_4_]∙5H_2_O			
C_20_H_41_N_3_O_21_Zn_2_	75–120.20	12.30(11.39)	5H_2_O
C_20_H_31_N_3_O_16_Zn_2_	120.20–190.55	10.76(9.12)	4 H_2_O
C_20_H_23_N_3_O_12_Zn_2_	190.55–360.35	29.23(29.87)	4 C_2_H_3_O_2_^−^
C_12_H_11_N_3_O_4_Zn_2_	360.35–Cont.	15.38(15.70)	C_6_H_6_O_2_N
C_6_H_5_N_2_O_2_Zn_2_			

[Cu_2_(L^2^)(OAc)_4_]∙2H_2_O∙2DMF shows two decomposition steps. The first decomposition step in the temperature range of 180.12–240.32 °C may be attributed to the loss of crystallization water molecules and two DMF molecules [36,37]. The second step within the temperature range of 240.32–285.34 °C 29.23(29.27%) corresponds to the removal of four moles of acetate molecules as deduced from weight loss calculations. The DTA curve of the Cu(II) complex shows two endothermic peaks at 160.50 °C and 190.10 °C. The endothermic peaks are likely due to the loss of water of hydration and DMF molecules.

The complex [Cu(L^2^)_2_]2NO_3_∙1.5DMF∙H_2_O is stable up to 100 °C and its decomposition started at this temperature. In the decomposition process of the Cu(II) complexes, the mass losses corresponded to one mole H_2_O, 1.5 moles DMF and two moles of NO_3_^−^ in the first stage of the decomposition, respectively. The Cu(II) complex is stable up to 160.00–240.00 °C and after this temperature other decomposition ensues. The DTA curve of the Cu(II) complex shows endothermic peaks at 100.20 °C and 215.50 °C corresponding to the loss of H_2_O, DMF and NO_3_^–^ and one exothermic peak at 500.10 °C corresponding to the second weight loss.

The [Zn_2_(L^2^)(OAc)_4_(H_2_O)_4_]∙5H_2_O complex followed a four-staged decomposition, in which five moles of water of crystallization, four moles of coordinated water, four moles of acetate ions, and an 2-iminocyclohexane-1,3-dione group and the rest of the ligand decomposed successively. The DTA curve of the Zn(II) complex shows two endothermic peaks at 130.50 °C and 180.10 °C. The endothermic peaks are likely due to the loss of molecules of water of hydration.

The decomposition curve of [Zn(L^2^)_2_(NO_3_)_2_]∙DMF begins with a step at 160.53–241.20 °C, displaying a 9.23(9.30%) weight loss corresponding to the removal of DMF [38]. The second step within the temperature range of 241.20–277.55 °C corresponds to the removal of two moles of NO_3_^−^ and C_12_H_14_N_2_O_2_ groups 44.61(44.10%) as deduced from weight loss calculations. The DTA curve of the Zn(II) complex shows one endothermic peak at 170.50 °C and two exothermic peaks at 300.60 °C and 350.40 °C. The first one is related to removal of the DMF molecule, whereas the other is the second step of decomposition [18].

The final products formed during thermal analysis of the complexes could not be determined because the decompositions of the complexes were not complete at 800 °C, except for the [Zn(L^1^)(NO_3_)_2_]∙4.5H_2_O and [Cu(L^2^)_2_]∙2NO_3_∙1.5DMF∙H_2_O complex.

### 2.5. Mass Spectra

The mass spectra peaks of the [Cu(L^1^)(OAc)_2_]∙5H_2_O, [Cu(L^1^)(NO_3_)H_2_O]∙NO_3_∙3.5H_2_O, [Zn(L^1^)(OAc)_2_(H_2_O)_2_]∙3H_2_O and [Zn(L^1^)(NO_3_)_2_]∙4.5H_2_O complexes of the L^1^ ligand are attributable to the related molecular ions *m*/*z*: 516.20 [M–H]^−^, 515.20 [M+H]^+^, 517.20 [M–2H]^2−^and 517.21 [M+H]^+^, respectively. The mass spectra peaks of the other L^2^ complexes [Cu_2_(L^2^)(OAc)_4_]∙2H_2_O∙2DMF, [Cu(L^2^)_2_]∙2NO_3_∙1.5DMF∙H_2_O, [Zn(L^2^)_2_(NO_3_)_2_]∙DMF and [Zn_2_(L^2^)(OAc)_4_(H_2_O)_4_]∙5H_2_O are attributable to the related molecular ions *m/z*: 807.21 [M+H]^+^, 838.14 [M+H]^+^, 785.32 [M+H]^+^ and 545.20 [M-2OAc^−^-7H_2_O]^2+^, respectively. The observed free ligand L^1^ peaks for all of the complexes are *m*/*z*: 247.11 [L+H]^+^ [38,39]. The observed free ligand L^2^ peaks for each two complexes are similarly *m*/*z*: 262.08 [L+H]^+^ except for the [Zn(L^2^)_2_(NO_3_)_2_]∙DMF complex. The highest peaks in the abundance percent were considered in the spectra.

**Figure 2:**
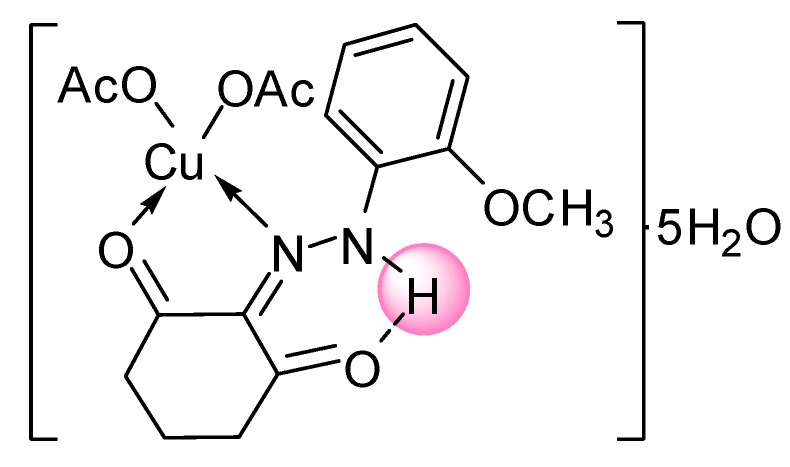
Structure of [(CuL^1^)(OAc)_2_]∙5H_2_O complex.

**Figure 3:**
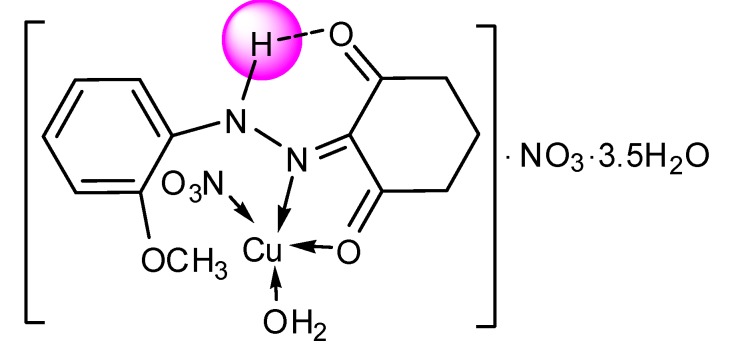
Structure of [Cu(L^1^)(NO_3_)H_2_O]NO_3_∙3.5H_2_O complex.

**Figure 4:**
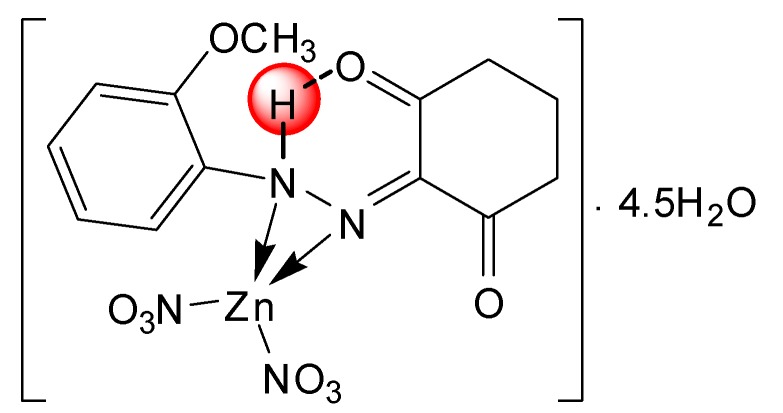
Structure of [Zn(L^1^)(NO_3_)_2_]∙4.5H_2_O complex.

**Figure 5:**
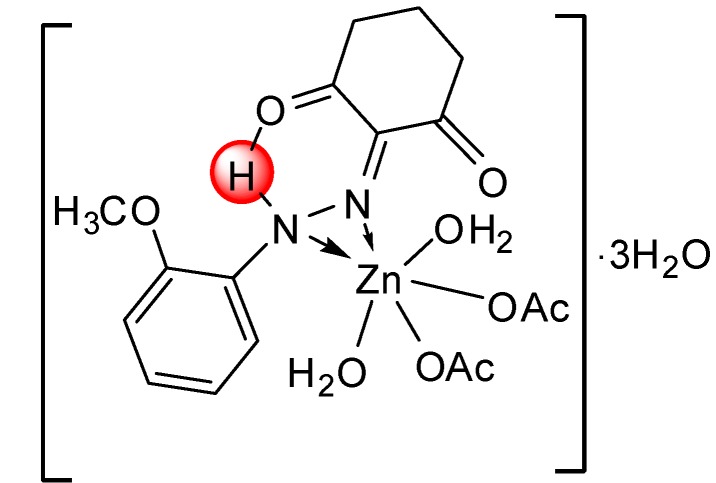
Structure of [Zn(L^1^)(OAc)_2_(H_2_O)_2_]∙3H_2_O complex.

**Figure 6:**
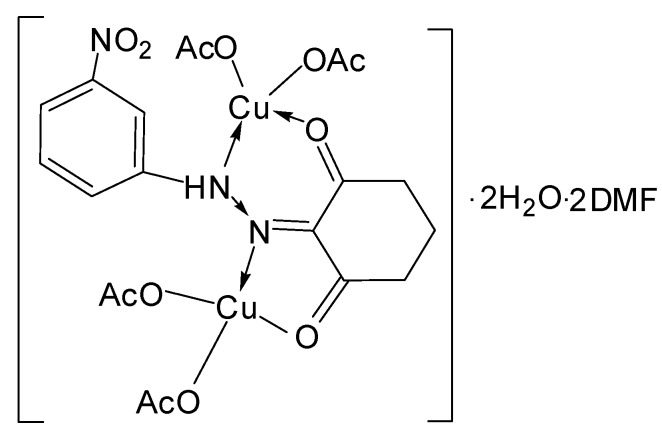
Structure of [Cu_2_(L^2^)(OAc)_4_]∙2H_2_O∙2DMF complex.

**Figure 7:**
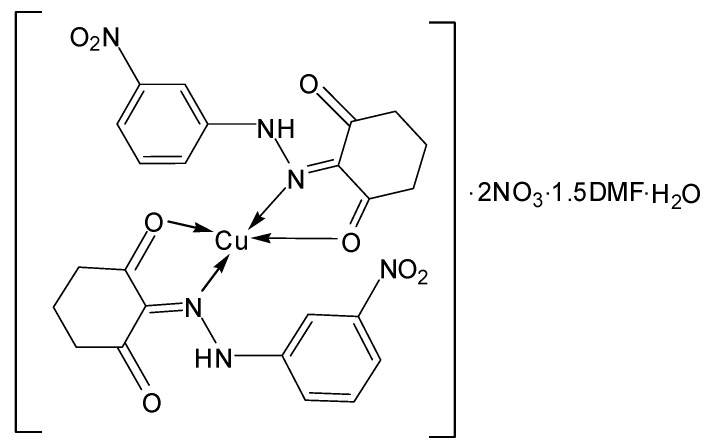
Structure of[Cu(L^2^)_2_]∙2NO_3_∙1.5DMF∙H_2_O complex.

**Figure 8:**
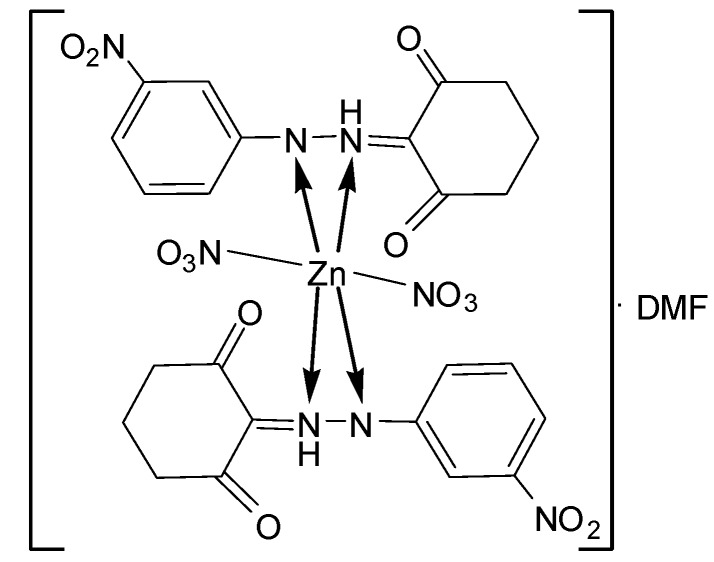
Structure of [Zn(L^2^)_2_(NO_3_)_2_]∙DMF complex.

**Figure 9:**
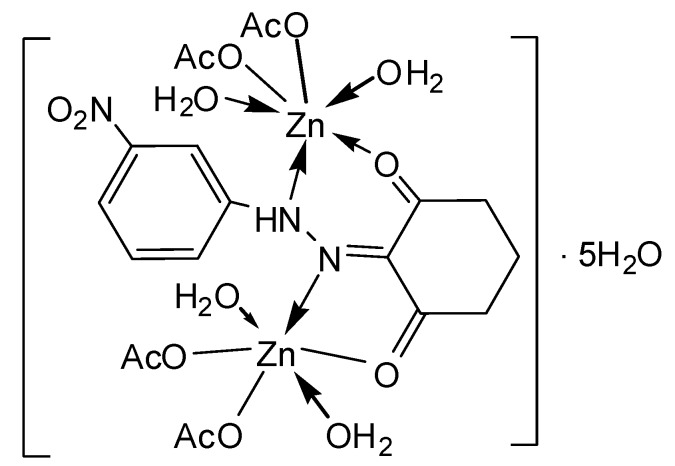
Structure of [Zn_2_(L^2^)(OAc)_4_(H_2_O)_4_]∙5H_2_O complex.

Single crystals of the complexes could not be isolated from any solutions, thus no definite structure could be described. However, the analytical, spectroscopic and magnetic data enable us to propose the possible structures shown below in Figure 2, Figure 3, Figure 4, Figure 5, Figure 6, Figure 7, Figure 8 and Figure 9.

### 2.6. Biological Evaluation

The synthesized compounds were screened for their antibacterial activity using *Escherichia coli* ATCC 25922, *Enterococcus faecalis* ATCC 29212, *Staphylococcus aureus* ATCC 25923, and *Salmonella typhimurium* CCM 583. The results are shown in Table 2. Control experiments were carried out under similar conditions using ampicillin as standard. The inhibition zone measurements in mm show that the compounds [Zn(L^1^)(OAc)_2_(H_2_O)_2_]∙3H_2_O, [Cu(L^2^)_2_]∙2NO_3_∙1.5DMF∙H_2_O and [Zn_2_(L^2^)(OAc)_4_(H_2_O)_4_]∙5H_2_O are more active than other tested compounds against the test bacteria. The other complexes [Zn(L^1^)(NO_3_)_2_]∙4.5H_2_O, [Cu(L^1^)(OAc)_2_]∙5H_2_O, [Cu(L^1^)(NO_3_)H_2_O]∙NO_3_∙3.5H_2_O, [Zn(L^2^)_2_(NO_3_)_2_]∙DMF and [Cu_2_(L^2^)(OAc)_4_]∙2H_2_O∙2DMF did not show antibacterial activity against test microorganisms like *Escherichia coli* ATCC 25922, *Enterococcus faecalis* ATCC 29212, *Staphylococcus aureus* ATCC 25923, and *Salmonella typhimurium* CCM 583.

**Table 2:** Antibacterial activity against test bacteria of the novel cyclohexane-1,3-dione ligands and their metal complexes.

Chemicals		*Escherichia coli* ATCC 25922	*Enterococcus faecalis* ATCC 29212	*Staphylococcus aureus* ATCC 25923	*Salmonella typhimurium* CCM 583
	Bacteria	Zone Diameter (mm) (blank disk diameter, 6 mm)			
[Cu(L^1^)(OAc)_2_]∙5H_2_O		0	0	0	0
[Cu(L^1^)(NO_3_)H_2_O]∙NO_3_∙3.5H_2_O		0	0	0	0
[Cu_2(_L^2^)(OAc)_4_]∙2H_2_O∙2DMF		0	0	0	0
[Cu(L^2^)_2_]∙2NO_3_∙1.5DMF∙H_2_O		0	0	11	7
[Zn(L^1^)(NO_3_)_2_]∙4.5H_2_O		0	0	0	0
[Zn(L^1^)(OAc)_2_(H_2_O)_2_]∙3H_2_O		10	0	11	8
[Zn(L^2^)_2_(NO_3_)_2_]∙DMF		0	0	0	0
[Zn_2_(L^2^)(OAc)_4_(H_2_O)_4_]∙5H_2_O		0	0	7	0
Ampicillin		16	22	32	26

## 3. Experimental Section

### 3.1. Reagents and Instrumentation

All solvents used were of analytical grade and no further purifications were performed. The metal salts Zn(NO_3_)_2_∙6H_2_O, Cu(NO_3_)_2_∙2H_2_O, Zn(OAc)_2_∙2H_2_O, Cu(OAc)_2_∙H_2_O and starting materials for the ligands were Merck (Darmstadt, Germany), Aldrich (St. Louis, MO, USA), and Alfa Aesar (Karlsruh, Germany) products.

Elemental analyses were carried out on a CHNS-O model 932 elemental analyzer (Leco, St. Joseph, MI, USA). ^1^H-NMR and ^13^C-NMR spectra were recorded using a model DPX-400 MHz FT spectrometer (Bruker GmbH, Billerica, MA, USA). IR spectra were recorded on a Precisely Spectrum One spectrometer (Perkin Elmer, Akron, OH, USA) using KBr discs in the wavenumber range of 4000–400 cm^−1^. Electronic spectra studies were conducted on a model UV-1700 spectrophotometer (Shimadzu, Kyoto, Japan) in the wavelength 1100–200 nm. Magnetic susceptibility measurements were performed using the standard Gouy tube technique. Hg[Co(SCN)_4_] was used for calibration calibrate. LC/MS-API-ES mass spectra were recorded using an Agilent model 1100 MSD mass spectrophotometer (Minneapolis, MN, USA). Thermogravimetric analysis (TGA) and differential thermal analysis (DTA) were carried out in nitrogen atmosphere with a heating rate of 10 °C∙min^−1^ using a Shimadzu DTG-60 AH thermal analyzer.

### 3.2. Antibacterial Activity Studies

The disk diffusion method was used for determining the antibacterial activity of ligands and complexes. Antibacterial activity against *Enterococcus faecalis* ATCC 29212, *Staphylococcus aureus* ATCC 25923, *Escherichia coli* ATCC 25922, and *Salmonella typhimurium* CCM 583 was investigated. Mueller–Hinton agar (Oxoid Ltd., Basingstoke, Hampshire, UK) was used for all bacterial strains, except for *Enterococcus faecalis* ATCC 29212 for which Mueller Hinton agar with 5% defibrinated sheep blood was used. The media were melted at 100 °C, autoclaved at 121 °C for 15 min, cooled 45 °C to 50 °C and were poured into plates of 9 cm diameter in quantities of 20 mL, and left on a flat surface to solidify and the surface of media was dried at 37 °C. Then, preparation of the inoculums was used for colony growth method in Mueller–Hinton broth to a turbidity equivalent to a 0.5 McFarland (10^8^ cfu∙mL^–1^) standard. The organisms were streaked on Petri dishes using sterile cotton swab. The surface of the media was allowed to dry 3–5 min at room temperature. The 10 mg∙mL^–1^ (in DMSO, Merck), of compound impregnated blank discs (Oxoid Ltd.) were applied to the surface of inoculated plates. The Mueller-Hinton agar plates were incubated at 35 ± 2 °C for 18–24 h. The plates were examined and the diameter of the inhibition zone was measured by surrounding discs. The antibiotic ampicillin (10 μg, Oxoid) was used as the standard [40,41].

### 3.3. Synthesis of the Ligands L^1^ and L^2^

A hydrochloric acid solution (2.5 mL) of 3-methoxyaniline (1.23 g, 10 mmol) and an aqueous solution (10 mL) of sodium nitrite (0.69 g, 10 mmol) were mixed and stirred at 273 K for 1 h. To this solution, an ethanol solution (10 mL) of the coupling component cyclohexane-1,3-dione (1.12 g, 10 mmol) was added and the stirring was continued at 273 K for 4 h. The resulting product was filtered and washed with water, dried and crystallized from ethanol (yield 77%). A similar synthesis was performed for L^2^ using 3-nitroaniline (1.38 g, 10 mmol) instead of 3-methoxyaniline.

**L^1^**; Yield: (77.0%). FW: 246.10 g∙mol^−1^. m.p.: 330 °C. Anal. Calcd. for C_13_H_14_N_2_O_3_: C, 63.40, H, 5.73, N, 11.38. Found: C, 64.00, H, 5.84, N, 11.49. Selected IR data (KBr, ν cm^−1^): 2955–3500 (N-H), 1696, 1671 (C=O), 1592 (C=N), 1505 (C=C), 1025 (N-N). UV-VIS (in DMF): λ_max_ (ε, L∙mol^−1^∙cm^−1^) 218, 242 (1453, 1613), 316–393 (2106–2620) nm; ^1^H-NMR (400 MHz, DMSO-*d_6_*): δ 15.00 (s, 1H, NH), 7.00–7.80 (m, 4H, Ar-CH), 2.00 (4H, CH_2_), 2.70 (2H, CH_2_), 4.00 (s, 3H, CH_3_). ^13^C-NMR (DMSO-*d_6_*): δ 30.12 (CH_3_), 55.87 (3CH_2_), 119.13 (Ar-H), 122.65 (Ar-H), 132.64 (Ar-NH-N), 147.00 (C=N), 197.00 (C=O), 197.10 (C=O). MS [ES]: *m*/*z* 247 [M+H]^+^. Color: Brown.

**L^2^**; Yield: (79.0%). FW: 261.10 g∙mol^−1^. m.p.: 332 °C. Anal. Calcd. for C_12_H_11_N_3_O_4_: C, 55.15, H, 4.21, N, 16.08. Found: C, 55.00, H, 4.84, N, 16.49. Selected IR data (KBr, ν cm^−1^): 3500–3100 (N-H), 1686 (C=O), 1614 (C=N), 1527 (C=C), 1010 (N-N). UV-VIS (in DMF): λ_max_ (ε, L∙mol^−1^∙cm^−1^) 218,240 (1453, 1612), 316–393 (2106–2620) nm; ^1^H-NMR (400 MHz, DMSO-*d_6_*): δ 14.56 (s, 1H, NH), 7.62–8.02 (m, 4H, Ar-CH), 2.60 (4H, CH_2_), 2.66 (2H, CH_2_). ^13^C-NMR (DMSO-*d_6_*): δ 30.12 (CH_2_), 52.37 (2CH_2_), 118.69 (Ar-H), 120.75 (Ar-H), 131.47 (Ar-NH-N), 143.05 (C=N), 193.37 (C=O), 197.55 (C=O). MS [ES]: *m*/*z* 262.08 [M+H]^+^. Color: Orange.

### 3.4. Synthesis of the Zn(II) and Cu(II) Complexes of the Ligands

**L^1^** (1 g, 4 mmol) was dissolved in absolute methanol (15 mL). A solution of 4.0 mmol of metal salts [Zn(NO_3_)_2_∙6H_2_O (1.19 g), Cu(NO_3_)_2_∙2H_2_O (0.94 g), Zn(OAc)_2_∙2H_2_O (0.88 g), and Cu(OAc)_2_∙H_2_O (0.80 g)] in an absolute mixture of methanol and DMF (10 mL) was added drop wise over 15 min. with continuous stirring at room temperature. The reaction mixtures were then further stirred for 6 h at 80 °C. The resulting precipitates were filtered, washed with absolute ether and dried at room temperature. Similar syntheses were performed for the L^2^ complexes using Zn(NO_3_)_2_∙6H_2_O (0.60 g, 2 mmol), Cu(NO_3_)_2_∙2H_2_O (0.48 g, 2 mmol), Zn(OAc)_2_∙2H_2_O (0.89 g, 4 mmol), and Cu(OAc)_2_∙H_2_O (1.60 g, 8 mmol), L^2^ (1 g, 4 mmol).

*[Cu(L^1^)(OAc)_2_]∙5H_2_O* (Figure 2): Yield: (78.0%). FW: 517.64 g∙mol^−1^. m.p.: >400 °C. μ_eff_(B.M.): 1.61. Anal. Calcd. for C_17_H_30_N_2_O_12_Cu: C, 39.41, H, 5.80, N, 5.41. Found: C, 39.50, H, 5.77, N, 5.39. Selected IR data (KBr, ν cm^−1^): 3468 (O-H), 3450 (N-H), 1665 (C=O), 1558 (C=N), 1507 (C=C), 1044 (N-N), 551–521 (M-O), 455–480 (M-N). UV-VIS (in DMF): λ_max_ (ε, L∙mol^−1^∙cm^−1^) 254,269 (1693–1793), 414 (266), 580 (386) nm; MS [ES]: *m*/*z* 516.64 (calc), 516.20 (found) [M–H]^–^, 247.10 (calc.), 247.11 (found) [L+H]^+^. Color: Dark black.

*[Cu(L^1^)(NO_3_)H_2_O]∙NO_3_∙3.5H_2_O* (Figure 3): Yield: (80.0%). FW: 514.64 g∙mol^−1^. m.p.: >390 °C. μ_eff_(B.M.): 1.72. Anal. Calcd. for C_13_H_16_N_4_O_10_Cu∙3.5H_2_O: C, 30.31, H, 4.47, N, 10.88. Found: C, 30.45, H, 4.55, N, 10.87. Selected IR data (KBr, ν cm^−1^): 3545 (O-H), 3313 (N-H), 1663 (C=O), 1593 (C=N), 1509 (C=C), 1041 (N-N), 585 (M-O), 491 (M-N). UV-VIS (in DMF): λ_max_ (ε, L∙mol^−1^∙cm^−1^) 254–295, 353, 393 (1693–1966, 2353, 262), 428–450 (285–300) nm; MS [ES]: *m*/*z* 515.64 (calc), 515.20 (found) [M+H]^+^, 247.10 (calc.), 247.11 (found) [L+H]^+^. Color: Dark green.

*[Zn(L^1^)(NO_3_)_2_]∙4.5H_2_O* (Figure 4): Yield: (75.0%). FW: 516.37 g∙mol^−1^. m.p.: 330 °C. μ_eff_(B.M.): Dia. Anal. Calcd. for C_13_H_14_N_4_O_9_Zn∙4.5H_2_O: C, 30.21, H, 4.45, N, 10.84. Found: C, 30.22, H, 4.38, N, 10.86. Selected IR data (KBr, ν cm^−1^): 3496 (O-H), 3317 (N-H), 1671 (C=O), 1590 (C=N), 1504 (C=C), 1042 (N-N), 543–514 (M-O), 475 (M-N). UV-VIS (in DMF): λ_max_(ε, L∙mol^−1^∙cm^−1^) 257–308 (1713–2053), 357 (2380) nm; MS [ES]: *m*/*z* 517.37 (calc), 517.21 (found) [M+H]^+^, 247.10 (calc.), 247.11 (found) [L+H]^+^. Color: Brown.

*[Zn(L^1^)(OAc)_2_(H_2_O)_2_]∙3H_2_O* (Figure 5): Yield: (75.0%). FW: 519.47 g∙mol^−1^. m.p.: 320 °C. μ_eff_(B.M.): Dia. Anal. Calcd. for C_17_H_30_N_2_O_12_Zn: C, 39.27, H, 5.78, N, 5.39. Found: C, 39.31, H, 5.76, N, 5.40. Selected IR data (KBr, ν cm^−1^): 3400 (O-H), Broad (N-H), 1671 (C=O), 1555 (C=N), 1506 (C=C), 1042 (N-N), 540–513 (M-O), 474 (M-N). UV-VIS (in DMF): λ_max_ (ε, L∙mol^−1^∙cm^−1^) 357–399 (2380–2660) nm; MS [ES]: *m*/*z* 517.47 (calc), 517.20 (found) [M–2H]^2−^,247.10 (calc.), 247.11 (found) [L+H]^+^. Color: Light brown.

*[Cu_2_(L^2^)(OAc)_4_]∙2H_2_O∙2DMF* (Figure 6): Yield: (76.0%). FW: 806.18 g∙mol^−1^. m.p.: >400. μ_eff_(B.M.): 1.66. Anal. Calcd. for C_26_H_41_N_5_O_16_Cu_2_: C, 38.70, H, 5.09, N, 8.68. Found: C, 38.57, H, 5.12, N, 8.73. Selected IR data (KBr, ν cm^−1^): 3436 (O-H), 3320 (N-H), 1660 (C=O), 1584 (C=N), 1528 (C=C), 1030 (N-N), 571–518 (M-O), 466 (M-N). UV-VIS (in DMF): λ_max_ (ε, L∙mol^−1^∙cm^−1^) 257–375 (1713–2500), 454 (302), 600 (66) nm; MS [ES]: *m*/*z* 807.18 (calc), 807.21 (found) [M+H]^+^, 542.18 (calc), 542.14 (found) [M-2AcO^−^-2H_2_O]^2+^ (Cationic complex) 262.10 (calc.), 262.08 (found) [L+H]^+^. Color: Dark brown.

*[Cu(L^2^)_2_]∙2NO_3_∙1.5DMF∙H_2_O* (Figure 7): Yield: (72.0%). FW: 837.24 g∙mol^−1^. m.p.: >400. μ_eff_(B.M.): 2.20. Anal. Calcd. for C_24_H_24_N_8_O_15_Cu∙1.5DMF: C, 40.85, H, 3.88, N, 15.89. Found: C, 40.86, H, 3.92, N, 15.91. Selected IR data (KBr, ν cm^−1^): 3441 (O-H), 3367 (N-H), 1663 (C=O), 1609 (C=N), 1528 (C=C), 1023 (N-N), 574–510 (M-O), 491–466 (M-N). UV-VIS (in DMF): λ_max_ (ε, L∙mol^−1^∙cm^−1^) 254–376 (1693–3760), 470 (313) nm; MS [ES]: *m*/*z* 838.24 (calc), 838.14 (found) [M+H]^+^, 574.74 (calc), 574.26 (found) [M-NO_3_^−^-1.5DMF-H_2_O]^+^ (Cationic complex) 262.10 (calc.), 262.08 (found) [L+H]^+^. Color: Green.

*[Zn(L^2^)_2_(NO_3_)_2_]∙DMF* (Figure 8): Yield: (68.0%). FW: 784.57 g∙mol^−1^. m.p.: 359. μ_eff_(B.M.): Dia. Anal. Calcd. for C_24_H_22_N_7_O_16_Zn: C, 41.30, H, 3.70, N, 14.28. Found: C, 40.33, H, 3.72, N, 14.26. Selected IR data (KBr, ν cm^−1^): 3300 (N-H), 1662 (C=O), 1606 (C=N), 1514 (C=C), 1020 (N-N), 580–520 (M-O), 473 (M-N). UV-VIS (in DMF): λ_max_ (ε, L∙mol^−1^∙cm^−1^) 357–375 (2380–2500) nm; MS [ES]: *m*/*z* 785.57 (calc), 785.32 (found) [M+H]^+^. Color: Dark brown.

*[Zn_2_(L^2^)(OAc)_4_(H_2_O)_4_]∙5H_2_O* (Figure 9): Yield: (64.0%). FW: 789.74 g∙mol^−1^. m.p.: 335. μ_eff_(B.M.): Dia. Anal. Calcd. for C_20_H_41_N_3_O_21_Zn_2_: C, 30.38, H, 5.19, N, 5.31. Found: C, 30.41, H, 5.20, N, 5.34. Selected IR data (KBr, ν cm^−1^): 3411 (O-H), (N-H)_broad_, 1668 (C=O), 1607 (C=N), 1528 (C=C), 1023 (N-N), 570–510 (M-O), 491–470 (M-N). UV-VIS (in DMF): λ_max_ (ε, L∙mol^−1^∙cm^−1^) 254–376 (1693–3760) nm; MS [ES]: *m*/*z* 545.84 (calc), 545.20 (found) [M-2AcO^−^-7H_2_O]^2+^ (Cationic complex) 262.10 (calc.), 262.08 (found) [L+H]^+^. Color: Black.

## 4. Conclusions

We have prepared two new ligands and their metal complexes. Structures of the ligands and complexes were confirmed by spectral and analytical techniques. Antibacterial activities of the complexes were tested against *Escherichia coli* ATCC 25922, *Enterococcus faecalis* ATCC 29212, *Staphylococcus aureus* ATCC 25923, and *Salmonella typhimurium* CCM 583, respectively. Some complexes showed average level antibacterial activity against the test bacteria compared to ampicillin, but some of the complexes did not exhibit antibacterial activity against the test microorganisms. For these reasons, these complexes may not be useful for preparing new active agents.

## References

[1] Lee D.L., Prisbylla M.P., Cromartie T.H., Dagarin D.P., Howard S.W., Provan W.M., Ellis M.K., Fraser T., Mutter L.C. (1997). The discovery and structural requirements of inhibitors of *p*-hydroxyphenylpyruvatedioxygene. Weed Sci..

[2] Lock E.A., Ellis M.K., Gaskin P., Robinson M., Auton T.R., Provan W.M., Smith L.L., Prisbylla M.P., Mutter L.C., Lee D.L. (1998). From toxicological problem to therapeutic use: the discovery of the mode of action of 2-(2-nitro-4-trifluoromethylbenzoyl)-1,3-cyclohexanedione (NTBC), its toxicology and development as a drug. J. Inherit. Metab. Dis..

[3] Sethukumar A., Arul Prakasam B. (2010). Spectral and cyclic voltammetric studies on some intramolecularly hydrogen bonded arylhydrazones: Crystal and molecular structure of 2-(2-(3-nitrophenyl)hydrazono)-5,5-dimethylcyclohexane-1,3-dione. J. Mol. Struct..

[4] Dey K. (1974). Schiff bases and their uses. J. Sci. Ind. Res..

[5] Nehru K., Athappan P., Rajagopal G. (2001). Ruthenium(II)/(III) complexes of bidentate acetyl hydrazide Schiff bases. Transit. Metal Chem..

[6] Deepa K., Aravindakshan K.K. (2005). Synthesis, characterization and antifungal studies of metal complexes of benzoyl- and salicylylhydrazones of ω-bromoacetoacetanilide. Synth. React. Inorg. Met..

[7] El-Dissouky A., Shuaib N.M., Al-Awadi N.A., Abbas A.B., El-Sherif A. (2008). Synthesis, characterization, potentiometric and thermodynamic studies of transition metal complexes with 1-benzotriazol-1-yl-1-[(*p*-methoxyphenyl)hydrazono]propan-2-one. J. Coord. Chem..

[8] Chang H-L., Chao T-Y., Yang C-C., Dai S.A., Jeng R.-J. (2007). Second-order nonlinear optical hyperbranched polymers via facile ring-opening addition reaction of azetidine-2,4-dione. Eur. Polym. J..

[9] Dabbagh H.A., Teimouri A. (2008). Insertion reaction of azidosulfonylazo dye with model alicyclic and heterocyclic compounds. Russ. J. Org. Chem..

[10] Tuncel M., Serin S. (2005). Synthesis and characterization of Copper(II), Nickel(II) and Cobalt(II) complexes with azo-linked Schiff base ligands. Synth. React. Inorg. Met..

[11] Raval J.P., Rai A.R., Patel N.H., Patel H.V., Patel P.S. (2009). Synthesis and *in vitro* antimicrobial activity of *N′*-(4-(arylamino)-6-(pyridin-2-ylamino)-1,3,5-triazin-2-yl)benzohydrazide. Int. J. ChemTech Res..

[12] Kuznik W., Kityk I.V., Kopylovich M.N., Mahmudov K.T., Ozga K., Lakshminarayana G., Pombeiro A.J.L. (2011). Quantum chemical simulations of solvent influence on UV-vis spectra and orbital shapes of azo derivatives of diphenylpropane-1,3-dione. Spectrochim. Acta Part A.

[13] Pulimamidi R.R., Addla S., Nomula R., Pallepogu R. (2011). Synthesis, structure, DNA binding and cleavage properties of ternary amino acid Schiff base-phen/bipy Cu(II) complexes. J. Inorg. Biochem..

[14] Bhowon M.G., Wah H.L.K., Dosieah A., Ridana M., Ramalingum O., Lacour D. (2004). Synthesis, characterization, and catalytic activity of metal Schiff base complexes derived from pyrrole-2-carboxyaldehyde. Synth. React. Inorg. Met..

[15] Berestovitskaya V.M., Selivanova M.V., Vakulenko M.I., Efremova I.E., Berkova G.A. (2009). Reactions of 2-benzylidene-3-methyl-4-nitro-2,5-dihydrothiophene 1,1-dioxide with CH acids. Russ. J. Org. Chem..

[16] Abu-El-Wafa S.M., El-Wakiel N.A., Issa R.M., Mansour R.A. (2005). Formation of novel mono- and multi-nuclear complexes of Mn(II), Co(II) and Cu(II) with bisazo-dianils containing the pyrimidine moiety: Thermal, magnetic and spectral studies. J. Coord. Chem..

[17] Anant P., Bibhesh K.S., Narendar B., Devjani A. (2010). Synthesis and Characterization of bioactive Zinc(II) and Cadmium(II) complexes with new Schiff bases derived from 4-nitrobenzaldehyde and acetophenone with ethylenediamine. Spectrochim. Acta Part A.

[18] Turan N., Sekerci M. (2009). Synthesis and spectral studies of novel Co(II), Ni(II), Cu(II), Cd(II), and Fe(II) metal complexes with *N*-[5′-amino-2,2′-bis(1,3,4-thiadiazole)-5-yl]-2-hydroxybenzaldehyde imine (HL). Spectrosc. Lett..

[19] El-Haty M.T., Adam F.A. (1986). Chelating properties of heterocyclic Schiff bases derived from 2-amino-5-phenyl-1,3,4-thiadiazole. Bull. Soc. Chim. Fr..

[20] Hui R-H., Zhou P., You Z.L. (2009). Syntheses crystal structures and antibacterial activities of two end-on azido bridged copper(II) complexes with Schiff bases. Indian J. Chem. Sect. A.

[21] Gup R., Kırkan B. (2005). Synthesis and spectroscopic studies of Copper(II) and Nickel(II) complexes containing hydrazonic ligands and heterocyclic coligand. Spectrochim. Acta Part A.

[22] Borras E., Alzuet G., Borras J., Serevr-Carrio J., Castineiras A., Liu-Gonzalez M., Sanz-Ruiz F. (2000). Coordination chemistry of sulfamethizole: Crystal structures of [Cu(sulfamethizolate)_2_(py)_2_(OH_2_)]H_2_O, [M(sulfamethizolate)_2_(py)_2_(OH_2_)_2_] [M = Co and Ni] and {Cu(sulfamethizolate)_2_(DMF)_2_}. Polyhedron.

[23] Cao G.-B. (2007). Synthesis, characterization, and crystal structure of a novel trinuclear Schiff base Nickel(II) complex. Synth. React. Inorg. Met..

[24] Chandra S., Vandana, Kumar S. (2015). Synthesis, spectroscopic, anticancer, antibacterial and antifungal studies of Ni(II) and Cu(II) complexes with hydrazine carboxyamide, 2-[3-methyl-2-thienylmethylene]. Spectrochim. Acta Part A.

[25] Şekerci M., Alkan C. (1999). The Synthesis and Co(II), Ni(II), Cu(II) and UO_2_(VI) complexes of 1,2-*O*-benzal-4-aza-7-aminoheptane. Synth. React. Inorg. Met..

[26] AbouEl-Enein S.A., El-Saied F.A., Kasher T.I., El-Wardany A.H. (2007). Synthesis and characterization of Iron(III), Manganese(II), Cobalt(II), Nickel(II), Copper(II) and Zinc(II) complexes of salicylidene-*N*-anilinoacetohydrazone (H_2_L^1^) and 2-hydroxy-1-naphthylidene-*N*-anilinoacetohydrazone (H_2_L^2^). Spectrochim. Acta Part A.

[27] Silverstein R.M., Bassler G.C., Morrill T.C. (1981). Spectrometric Identification of Organic Compounds.

[28] Losada J., delPeso I., Beyer L. (2001). Electrochemical and spectroelectrochemical properties of Copper(II) Schiff-base complexes. Inorg. Chim. Acta.

[29] Tabassum S., Bashar A., Arjmand F., Siddigi G. (1997). Synthesis and characterization of lanthanide chelates with metal-containing ligands. Synth. React. Inorg. Met..

[30] Nakamoto K. (1970). Infrared Spectra of Inorganic and Coordination Compounds.

[31] Tharmaraj P., Kodimunthiri D., Prakash P., Sheela C.D. (2009). Catalytic and biological activity of transition metal complexes of salicylaldiminopropylphosphine. J. Coord. Chem..

[32] Uçan S.Y., Uçan M. (2005). Synthesis and characterization of new Schiff bases and their Cobalt(II), Nickel(II), Copper(II), Zinc(II), Cadmium(II) and Mercury(II) complexes. Synth. React. Inorg. Met..

[33] El-Metwally N.M., Gabr I.M., Shallaby A.M., El-Asmy A.A. (2005). Synthesis and spectroscopic characterization of new mono- and binuclear complexes of some NH(1) thiosemicarbazides. J. Coord. Chem..

[34] Sönmez M., Sekerci M. (2004). The template synthesis, characterization, and thermal investigation of new heterocyclic binucleating Schiff base complexes. Synth. React. Inorg. Met..

[35] Sekerci M. (2000). Synthesis, magnetospectral, and thermal studies of Cobalt(II), Nickel(II), and Copper(II) Complexes of new asymmetrical 1,2-dihydroxyimino-3,8-diaza-9-phenylnonane. Russ. J. Inorg. Chem..

[36] Turan N., Sekerci M. (2010). Synthesis, characterization and thermal behavior of some Zn(II) complexes with ligands having 1,3,4-thiadiazole moieties. Heteroat. Chem..

[37] El-Bindary A.A., El-Sonbati A.Z., Diab M.A., Attallah M.E. (2012). Polymer complexes. LVI. supramolecular architectures consolidated by hydrogen bonding and π–π Interaction. Spectrochim. Acta Part A.

[38] Adiguzel R., Esener H., Ergin Z., Aktan E., Turan N., Sekerci M. (2011). Synthesis and structural characterization of bis(2-amino-1,3,4-thiadiazolyl)methane complexes. Asian J. Chem..

[39] Esener H., Adıgüzel R., Ergin Z., Aktan E., Turan N., Sekerci M. (2011). Synthesis and characterization of novel Mn(II), Co(II), Ni(II) and Cd(II) complexes from 4-(2-nitrophenylazo)-1*H*-pyrazole-3,5-diamine. Adv. Sci. Lett..

[40] Clinical and Laboratory Standards Institute (2006). Performance Standards for Antimicrobial Disk Susceptibility Tests.

[41] National Committee for Clinical Laboratory Standards (2000). Performance Standards for Antimicrobial Disk Susceptibility Tests.

